# Supplementary material for: Effectiveness and Components of Web-Based Interventions on Weight Changes in Adults Who Were Overweight and Obese: A Systematic Review with Meta-Analyses
Source: Nutrients. 2022 Dec 30;15(1):179. doi: 10.3390/nu15010179 (PMC9823835; doi:10.3390/nu15010179)
Supplement: Supplementary file 1 [file nutrients-15-00179-s001.zip › nutrients-2069481-supplementary.pdf]

Supplementary Table S1. The search terms and formulas for PubMed.

	MeSH/Limits	Other key words	Search query	Search #
population	overweight	obesity	overweight[mh] OR obesity[tiab]	#1
intervention	internet-based intervention	smartphone*<br>mobile*<br>application*<br>technology<br>internet<br>computer*<br>social media<br>online system*	"internet-based intervention"[mh] OR smartphone*[tiab] OR mobile*[tiab] OR application*[tiab] OR technology[tiab] OR internet[tiab] OR computer*[tiab] OR "social media"[tiab] OR "online system*"[tiab]	#2
publication type	N/A	randomized controlled trial<br>controlled clinical trial<br>random*<br>placebo<br>trial*<br>group*<br>program*	"randomized controlled trial"[pt] OR "controlled clinical trial"[pt] OR random*[all fields] OR placebo[tiab] OR trial*[tiab] OR group*[tiab] OR program*[tiab]	#3
outcome	body weight changes	weight loss<br>weight maintenance<br>weight reduction	"body weight changes"[mh] OR "weight loss"[tiab] OR "weight maintenance"[tiab] OR "weight reduction"[tiab]	#4

N/A, not available; RCT, randomized controlled trial; [mh], MeSH Terms; [pt], publication type; [tiab], title and abstract; #1AND#2AND#3AND#4=1305（Date of search：September 30, 2020）

Supplementary Table S2. The search terms and formulas for Ichu-shi Web.

	Key concept	Thesauri	Other keywords	Search Query	Search #
publication type	original articles	original articles	N/A	PT＝original articles	#1
intervention	ウェブベース介入 (web-based intervention)	インターネット(internet)<br>スマートフォン (smartphone)	インターネット(internet)<br>internet<br>ウェブ(web)<br>web<br>モバイル(mobile)<br>mobile<br>アプリケーション (application)<br>アプリ(apps)<br>apps<br>application<br>スマートフォン (smartphone)<br>smartphone	(インターネット/TH OR インターネット/AL) OR (インターネット/TH OR internet/AL) OR (ウェブ/AL OR web/AL) OR (モバイル/AL OR mobile/AL) OR (アプリケーション/AL OR アプリ/AL) OR (apps/AL OR application/AL) OR (スマートフォン/TH OR スマートフォン/AL) OR (スマートフォン/TH OR smartphone/AL)	#2
outcome	体重変化(weight change)	体重(weight)<br>体重減少(weight loss)<br>体重変化(weight change)<br>肥満(obesity)<br>やせ(thin)<br>BMI<br>体重減少プログラム (weight loss program)	N/A	体重/TH OR 体重減少/TH OR 体重変化/TH OR 肥満/TH OR やせ/TH OR BMI/TH OR 体重減少プログラム/TH	#3

AL, all fields; BMI, body mass index; N/A, not available; PT, publication type; TH, thesaurus; #1AND#2AND#3=160 (Date of search：September 30, 2020)

Supplementary Table S3. The specific features of the retrieved studies.

Title	Author	Publication year	Setting	Intervention device	Male%	Retention rate	Mean BMI	Mean age	Intervention period	Effectiveness
Computerized weight loss intervention optimizes staff time: the clinical and cost results of a controlled clinical trial conducted in a managed care setting	Wylie-Rosett J et al.	2001	USA	computer	17.7%	81.0%	35.6	52.2	12 months	Yes
Effects of Internet behavioral counseling on weight loss in adults at risk for type 2 diabetes: a randomized trial	Tate DF et al.	2003	USA	computer	10.9%	83.7%	33.1	48.5	12 months	Yes
A randomized controlled trial of a commercial internet weight loss program	Womble LG et al.	2004	USA	computer	0.0%	66.0%	33.5	43.7	12 months	No
Web-based weight management programs in an integrated health care setting: a randomized, controlled trial	Rothert K et al.	2006	USA	computer	17.0%	70.0%	32.1	45.4	6 months	Yes
A randomized controlled trial on the long-term effects of a 1-month behavioral weight control program assisted by computer tailored advice	Adachi Y et al.	2007	Japan	computer	0.0%	75.6%	26.1	46.2	7 months	Yes
Weight loss on the web: A pilot study comparing a structured behavioral intervention to a commercial program	Gold BC et al.	2007	USA	computer	18.5%	73.4%	32.4	47.7	6 months	Yes
The Internet for weight control in an obese sample: results of a randomised controlled trial	McConnon A et al.	2007	UK	computer	N/A	59.0%	34.4	45.8	12 months	No
Minimal in-person support as an adjunct to internet obesity treatment	Micco N et al.	2007	USA	computer	17.1%	79.0%	31.7	46.8	12 months	No
Motivational interviewing in internet groups: a pilot study for weight loss	Webber Kh et al.	2008	USA	computer	N/A	56.3%	31.0	40.6	2 months	Yes
Comparison of methods for delivering a lifestyle modification program for obese patients: a randomized trial	Digenio AG et al.	2009	USA	computer	13.0%	70.0%	34.4	43.8	6 months	No
The SHED-IT randomized controlled trial: evaluation of an Internet-based weight-loss program for men	Morgan PJ et al.	2009	Australia	computer	100.0%	83.0%	30.6	35.9	6 months	No
Pounds Off Digitally study: a randomized podcasting weight-loss intervention	Turner-McGrievy GM et al.	2009	USA	computer	25.6%	83.0%	31.6	38.6	3 months	No
Phone and e-mail counselling are effective for weight management in an overweight working population: a randomized controlled trial	van Wier MF et al.	2009	Netherlands	computer	67.0%	70.9%	29.6	43.0	6 months	Yes
Internet delivered behavioral obesity treatment	Harvey-Berino J et al.	2010	USA	computer	7.0%	96.0%	35.7	46.6	6 months	No
Treating obesity with a novel hand-held device, computer software program, and Internet technology in primary care: the SMART motivational trial	McDoniel SO et al.	2010	USA	computer	39.0%	72.0%	37.0	45.5	3 months	No
Effects of a non-face-to-face behavioral weight-control program among Japanese overweight males: a randomized controlled trial	Tanaka M et al.	2010	Japan	computer	0.0%	90.2%	26.2	46.0	7 months	No

Motivation and its relationship to adherence to self-monitoring and weight loss in a 16-week Internet behavioral weight loss intervention	Webber KH et al.	2010	USA	computer	0.0%	98.0%	31.1	50.1	4 months	No
Effect of an individually tailored one-year energy balance programme on body weight, body composition and lifestyle in recent retirees: a cluster randomised controlled trial	Werkman A et al.	2010	Netherlands	computer	85.2%	93.9%	27.0	59.5	12 months	No
The effect of electronic self-monitoring on weight loss and dietary intake: a randomized behavioral weight loss trial	Burke LE et al.	2011	USA	computer	15.0%	91.0%	34.1	46.8	6 months	No
Men participating in a weight-loss intervention are able to implement key dietary messages, but not those relating to vegetables or alcohol: the Self-Help, Exercise and Diet using Internet Technology (SHED-IT) study	Collins CE et al.	2011	Australia	computer	100.0%	82.0%	30.6	35.9	6 months	No
Directive and nondirective e-coach support for weight loss in overweight adults	Gabriele JM et al.	2011	USA	computer	16.3%	93.3%	32.1	45.4	3 months	No
Primary care referral to a commercial provider for weight loss treatment versus standard care: a randomised controlled trial	Jebb SA et al.	2011	UK	computer	13.5%	57.5%	31.4	47.4	12 months	Yes
12-month outcomes and process evaluation of the SHED-IT RCT: an internet-based weight loss program targeting men	Morgan PJ et al.	2011	Australia	computer	100.0%	71.0%	30.6	35.9	12 months	No
Outcomes of a 12-month web-based intervention for overweight and obese men	Patrick K et al.	2011	USA	computer	N/A	70.0%	N/A	43.9	12 months	No
Effectiveness of phone and e-mail lifestyle counseling for long term weight control among overweight employees	van Wier MF et al.	2011	Netherlands	computer	67.0%	54.5%	29.6	43.0	6 months	Yes
Use of a computerized tracking system to monitor and provide feedback on dietary goals for calorie-restricted diets: the POUNDS LOST study	Anton SD et al.	2012	USA	computer	37.6%	N/A	N/A	N/A	24 months	N/A
Features predicting weight loss in overweight or obese participants in a web-based intervention: randomized trial	Brindal E et al.	2012	Australia	computer	16.6%	16.4%	34.0	45.0	3 months	No
Economic evaluation of a weight control program with e-mail and telephone counseling among overweight employees: a randomized controlled trial	van Wier MF et al.	2012	Netherlands	computer	67.0%	55.0%	29.6	43.0	24 months	No
Randomized Controlled Pilot Study Testing Use of Smartphone Technology for Obesity Treatment	Allen JK et al.	2013	USA	smartphone	22.1%	63.2%	34.3	44.9	6 months	No

Design and pilot results of a mobile phone weight-loss application for women starting a meal replacement programme	Brindal E et al.	2013	Australia	smartphone	0.0%	76.0%	34.0	42.0	2 months	No
Randomized trial of nutrition education added to internet-based information and exercise at the work place for weight loss in a racially diverse population of overweight women	Carnie A et al.	2013	USA	computer	0.0%	69.8%	33.9	46.0	6 months	No
Adherence to a Smartphone Application for Weight Loss Compared to Website and Paper Diary: Pilot Randomized Controlled Trial	Carter MC et al.	2013	UK	smartphone	23.0%	61.7%	34.0	42.0	6 months	No
Efficacy of Standard Versus Enhanced Features in a Web-Based Commercial Weight-Loss Program for Obese Adults,Part 2: Randomized Controlled Trial	Collins CE et al.	2013	Australia	computer	41.7%	97.4%	32.2	42.0	6 months	No
Cost-effectiveness of a distance lifestyle counselling programme among overweight employees from a company perspective, ALIFE@Work: a randomized controlled trial	Gussenhoven AH et al.	2013	Netherlands	computer	N/A	65.9%	29.2	N/A	24 months	No
Effectiveness of Web-Based Self-Disclosure Peer-to-Peer Support for Weight Loss: Randomized Controlled Trial	Imanaka M et al.	2013	Japan	computer	86.0%	90.7%	27.5	50.0	3 months	Yes
A Randomized Controlled Trial of a Community-based Behavioral Counseling Program	Johnston CA et al.	2013	USA	computer & smartphone	10.2%	88.0%	33.0	46.5	6 months	Yes
The SHED-IT community trial: a randomized controlled trial of internet- and paper-based weight loss programs tailored for overweight and obese men	Morgan PJ et al.	2013	Australia	computer	100.0%	81.0%	32.7	47.5	6 months	Yes
Using Facebook and text messaging to deliver a weight loss program to college students	Napolitano MA et al.	2013	USA	smartphone	13.5%	100.0%	31.4	20.5	2 months	Yes
Integrating technology into standard weight loss treatment: a randomized controlled trial	Spring B et al.	2013	USA	smartphone	85.5%	81.4%	36.3	57.7	12 months	Yes
Comparison of traditional versus mobile app self-monitoring of physical activity and dietary intake among overweight adults participating in an mHealth weight loss program	Turner-McGrievy GM et al.	2013	USA	smartphone	23.9%	90.0%	32.5	44.0	6 months	Yes
A pilot Internet-based behavioral weight loss intervention with or without commercially available portion-controlled foods	Webber KH et al.	2013	USA	computer	14.0%	94.0%	35.1	46.0	3 months	Yes
A Randomized Controlled Trial Comparing Scalable Weight Loss Treatments in Primary Care	Barnes RD et al.	2014	USA	computer	23.6%	96.6%	35.3	47.9	3 months	Yes

Teledietetics Improves Weight Reduction by Modifying Eating Behavior: A Randomized Controlled Trial	Chung LM et al.	2014	Hong Kong	computer	36.7%	90.0%	27.8	37.4	3 months	Yes
Does Brief Telephone Support Improve Engagement With a Web-Based Weight Management Intervention?Randomized Controlled Trial	Dennison L et al.	2014	UK	computer	20.1%	32.4%	33.0	44.0	2 months	No
Effectiveness of a smartphone application for weight loss compared to usual care in overweight primary care patients: a randomized controlled trial	Laing BY et al.	2014	USA	smartphone	27.0%	74.1%	33.4	43.3	6 months	No
Associations between program outcomes and adherence to Social Cognitive Theory tasks: process evaluation of the SHED-IT community weight loss trial for men	Morgan PJ et al.	2014	Australia	computer	100.0%	66.0%	32.6	47.3	6 months	No
Impact of visceral fat measurements and a weight loss support web system on visceral fat loss in a workplace setting: insights from a JVALUE2 (In Japanese)	Okazaki H et al.	2014	Japan	computer	91.0%	90.0%	26.4	46.2	3 months	Yes
Effect of the Intervention Based on New Communication Technologies and the Social-Cognitive Theory on the Weight Control of the Employees with Overweight and Obesity	Abdi J et al.	2015	Iran	computer	28.7%	N/A	N/A	42.0	6 months	Yes
Virtual small groups for weight management: an innovative delivery mechanism for evidence-based lifestyle interventions among obese men	Azar KM et al.	2015	USA	computer	100.0%	75.0%	34.8	46.3	3 months	Yes
Diabetes Prevention and Weight Loss with a Fully Automated Behavioral Intervention by Email, Web, and Mobile Phone: A Randomized Controlled Trial Among Persons with Prediabetes	Block G et al.	2015	USA	smartphone	68.7%	N/A	31.1	55.0	6 months	Yes
A Randomized Trial Testing the Efficacy of a Novel Approach to Weight Loss Among Men With Overweight and Obesity	Crane MM et al.	2015	USA	computer	100.0%	90.7%	31.5	44.2	6 months	Yes
A Novel Diabetes Prevention Intervention Using a Mobile App: A Randomized Controlled Trial With Overweight Adults at Risk	Fukuoka Y et al.	2015	Japan	smartphone	23.0%	91.8%	33.3	55.2	5 months	Yes
Benefits of adding Small Financial Incentives or Optional Group Meetings to a Web-based Statewide Obesity Initiative	Leahey TM et al.	2015	USA	computer	17.5%	91.4%	33.6	46.3	3 months	No
Efficacy of SmartLoss℠, a smartphone-based weight loss intervention: Results from a randomized controlled trial	Martin CK et al.	2015	USA	smartphone	17.5%	95.0%	29.8	44.4	3 months	Yes
The Effectiveness of Mobile Phone-Based Care for Weight Control in Metabolic Syndrome Patients: Randomized Controlled Trial	Oh B et al.	2015	Korea	smartphone	50.9%	79.1%	29.4	48.6	6 months	Yes
Cell phone Intervention for You (CITY): A randomized, controlled trial of behavioral weight loss intervention for young adults using mobile technology	Svetkey LP et al.	2015	USA	smartphone	30.4%	86.0%	35.2	29.4	24 months	No
An Automated Internet Behavioral Weight-Loss Program by Physician Referral: A Randomized Controlled Trial	Thomas JG et al.	2015	USA	computer	20.0%	84.4%	34.9	53.2	3 months	Yes

A preliminary investigation into whether early intervention can improve weight loss among those initially non-responsive to an internet-based behavioral program	Unick JL et al.	2015	USA	computer	8.0%	92.0%	33.4	51.7	3 months	No
Effect of a Web-Based Behavior Change Program on Weight Loss and Cardiovascular Risk Factors in Overweight and Obese Adults at High Risk of Developing Cardiovascular Disease: Randomized Controlled Trial	Watson S et al.	2015	UK	computer	44.6%	70.8%	32.7	52.1	12 months	Yes
Using Social and Mobile Tools for Weight Loss in Overweight and Obese Young Adults (Project SMART): A 2-Year Parallel Group Randomized Controlled Trial	Godino JG et al.	2016	USA	computer	29.7%	84.4%	29.0	22.7	24 months	Yes
Social networks for improving healthy weight loss behaviors foroverweight and obese adults: A randomized clinical trial of the socialpounds off digitally (Social POD) mobile app	Hales S et al.	2016	USA	smartphone	18.0%	82.4%	34.7	46.2	3 months	Yes
Effect of Wearable Technology Combined with a Lifestyle Intervention on Long-Term Weight Loss: the IDEA Randomized Clinical Trial	Jakicic JM et al.	2016	USA	computer	28.9%	74.5%	31.2	30.9	24 months	Yes
An internet-based intervention with brief nurse support to manage obesity in primary care (POWeR+): a pragmatic, parallel-group, randomised controlled trial	Little P et al.	2016	UK	computer	63.6%	80.6%	36.7	53.7	12 months	No
An Internet-Assisted Weight Loss Intervention for Older Overweight and Obese Rural Women A Feasibility Study	O'Brien T et al.	2016	USA	computer	0.0%	87.5%	34.2	69.0	3 months	No
Weight Control Intervention for Truck Drivers: The SHIFT Randomized Controlled Trial, United States	Olson R et al.	2016	Poland	computer	86.1%	80.1%	35.6	47.8	6 months	Yes
A Comparative Effectiveness Trial of Three Walking Self-monitoring Strategies	Richardson CR et al.	2016	USA	computer	N/A	72.0%	36.3	56.3	6 months	Yes
Do Individual Online Motivational Interviewing Chat Sessions Enhance Weight Loss in a Group Online Weight Control Program?	West DS et al.	2016	USA	computer	10.3%	81.0%	36.0	48.4	18 months	No
A Feasible and Efficacious Mobile-Phone Based Lifestyle Intervention for Filipino Americans with Type 2 Diabetes: Randomized Controlled Trial	Bender MS et al.	2017	USA	smartphone	38.0%	100.0%	30.1	57.6	3 months	Yes
The SMARTER pilot study: Testing feasibility of real-time feedback for dietary self-monitoring	Burke LE et al.	2017	USA	smartphone	12.8%	74.4%	33.8	44.9	3 months	No
Personalized Mobile Health Intervention for Health and Weight Loss in Postpartum Women Receiving Women, Infants, and Children Benefit: A Randomized Controlled Pilot Study	Gilmore LA et al.	2017	USA	smartphone	0.0%	87.5%	26.5	26.0	4 months	No

Effects of a weight management program delivered by social media on weight and metabolic syndrome risk factors in overweight and obese adults: A randomised controlled trial	Jane M et al.	2017	Australia	computer	14.9%	44.5%	37.9	50.4	3 months	Yes
Randomised controlled trial and economic analysis of an internet-based weight management programme: POWeR+ (Positive Online Weight Reduction)	Little P et al.	2017	UK	smartphone	36.4%	80.6%	36.7	53.7	12 months	No
Internet-delivered obesity treatment improves symptoms of and risk for depression	Naparstek J et al.	2017	USA	computer	18.4%	N/A	34.9	46.9	3 months	Yes
Hockey Fans in Training: A Pilot Pragmatic Randomized Controlled Trial	Petrella RJ et al.	2017	Canada	computer	100.0%	93.8%	36.5	48.7	12 months	Yes
Effect of an Internet-Based Program onWeight Loss for Low-Income PostpartumWomen A Randomized Clinical Trial	Phelan S et al.	2017	USA	computer	0.0%	81.9%	31.7	28.1	12 months	Yes
Effects of an Abbreviated Obesity Intervention Supported by Mobile Technology: The ENGAGED Randomized Clinical Trial	Spring B et al.	2017	USA	smartphone	16.0%	86.5%	34.6	39.3	6 months	Yes
Weight loss and frequency of body-weight self-monitoring in an online commercial weight management program with and without a cellular-connected 'smart' scale: a randomized pilot study	Thomas JG et al.	2017	USA	computer	16.3%	94.6%	34.0	55.6	6 months	No
Improving Weight in People with Serious Mental Illness: The Effectiveness of Computerized Services with Peer Coaches	Young AS et al.	2017	USA	computer	N/A	N/A	N/A	N/A	6 months	No
Filipinos Fit and Trim - A feasible and efficacious DPP-based intervention trial	Bender MS et al.	2018	USA	computer	32.0%	91.0%	30.5	41.7	3 months	Yes
Short- and long-term effectiveness of a smartphone application for improving measures of adiposity: A randomised clinical trial – EVIDENT II study	Gomez-Marcos MA et al.	2018	Spain	smartphone	37.9%	85.8%	27.8	51.9	3 months	No
Psychological effects of belonging to a Facebook weight management group in overweight and obese adults: Results of a randomised controlled trial	Jane M et al.	2018	Australia	computer	N/A	39.4%	N/A	N/A	6 months	Yes
A randomized clinical trial of the effectiveness of a Web-based health behaviour change support system and group lifestyle counselling on body weight loss in overweight and obese subjects: 2-year outcomes	Teeriniemi AM et al.	2018	Finland	computer	50.9%	70.5%	30.5	46.0	24 months	No
A Behavioral Lifestyle Intervention Enhanced With Multiple-Behavior Self-Monitoring Using Mobile and Connected Tools for Underserved Individuals With Type 2 Diabetes and Comorbid Overweight or Obesity: Pilot Comparative Effectiveness Trial	Wang J et al.	2018	USA	smartphone	38.0%	92.0%	38.1	56.4	6 months	No

The efficacy of a telemedicine-based weight loss program with video conference health coaching support	Alencar MK et al.	2019	USA	computer	48.0%	83.3%	34.7	46.6	3 months	Yes
Effectiveness of randomized controlled trial of a mobile app to promote healthy lifestyle in obese and overweight patients	Apiñaniz A et al.	2019	Spain	smartphone	28.2%	60.0%	32.7	38.5	6 months	No
Dietary Self-Monitoring Through Calorie Tracking but Not Through a Digital Photography App Is Associated with Significant Weight Loss: The 2SMART Pilot Study—A 6-Month Randomized Trial	Dunn CG et al.	2019	USA	smartphone	9.3%	69.8%	34.5	42.4	6 months	No
Randomized controlled trial of OnTrack, a just-in-time adaptive intervention designed to enhance weight loss	Forman EM et al.	2019	USA	computer	14.9%	88.4%	34.3	46.3	2.5 months	No
Evaluating differences in the clinical impact of a free online weight loss programme, a resource-intensive commercial weight loss programme and an active control condition: a parallel randomised controlled trial	Innes AQ et al.	2019	UK	computer	34.2%	82.9%	35.4	38.3	3 months	Yes
Development of the Top Tips Habit-Based Weight Loss App and Preliminary Indications of Its Usage, Effectiveness, and Acceptability: Mixed-Methods Pilot Study	Kliemann N et al	2019	France	computer	10.0%	65.5%	34.3	42.4	3 months	Yes
Randomized controlled trial of Web - based weight - loss intervention with human support for male workers under 40	Ozaki I et al.	2019	Japan	smartphone	100.0%	88.8%	28.7	34.2	3 months	Yes
Comparing Self-Monitoring Strategies for Weight Loss in a Smartphone App: Randomized Controlled Trial	Patel ML et al.	2019	USA	smartphone	16.0%	72.4%	31.9	42.7	3 months	No
Comparison of Smartphone-based Behavioral Obesity Treatment to Gold Standard Group Treatment and Control: A Randomized Trial	Thomas JG et al.	2019	USA	smartphone	17.0%	78.6%	35.2	55.1	18 months	No
A technology-assisted health coaching intervention vs. enhanced usual care for Primary Care-Based Obesity Treatment: a randomized controlled trial	Viglione C et al.	2019	USA	computer	67.4%	84.4%	32.0	54.5	12 months	No
Enhancing group - based internet obesity treatment: A pilot RCT comparing video and text - based chat	West DS et al.	2019	USA	computer	0.0%	75.0%	34.1	47.2	6 months	No
Testing an Internet-Based Turkish Obesity Behavioral Therapy Program: A Randomized Study	Hepdurgun C et al.	2020	Turkey	computer	20.8%	75.2%	31.9	40.6	2 months	Yes
Impact of a Web-Based Exercise and Nutritional Education Intervention in Patients Who Are Obese With Hypertension: Randomized Wait-List Controlled Trial	Lisón JF et al.	2020	Spain	computer	N/A	90.5%	30.0	53.2	3 months	Yes
Adding Financial Incentives to Online Group-Based Behavioral Weight Control: An RCT	West DS et al.	2020	USA	computer	8.9%	86.1%	35.7	49.0	6 months	No

Supplementary Table S4. The quality of the evidence of 97 articles.

Authors (publication year)	Study design	Random sequence generation	Allocation concealment	Blinding of participants and personnel	Blinding of outcome assessment	Incomplete outcome data	Selective reporting	Other bias	Summary
Wylie-Rosett J et al. (2001)	RCT	−1	−1	−1	−1	0	−1	0	−1
Tate DF et al. (2003)	RCT	0	0	−1	−1	0	−1	0	0
Womble LG et al. (2004)	RCT	−1	−1	−1	−1	0	−1	0	−1
Rothert K et al. (2006)	RCT	−1	0	−1	−1	0	−1	0	0
Adachi Y et al. (2007)	RCT	−1	−1	−1	−1	0	−1	0	−1
Gold BC et al. (2007)	RCT	−1	−1	−1	−1	0	−1	0	−1
Micco N et al. (2007)	RCT	−1	−1	−1	−1	0	−1	0	−1
McConnon A et al. (2007)	RCT	0	−1	−2	−2	0	0	0	−1
Webber Kh et al. (2008)	RCT	−1	−1	−1	−1	0	−1	0	−1
van Wier MF et al. (2009)	RCT	0	0	−1	−1	0	0	−2	−1
Digenio AG et al. (2009)	RCT	0	−1	−1	−1	0	−1	−1	−1
Morgan PJ et al. (2009)	RCT	0	0	0	0	0	0	0	0
Turner-McGrievy GM et al. (2009)	RCT	−1	−1	−1	−1	0	−1	0	−1
Tanaka M et al. (2010)	RCT	−1	−1	−1	−1	0	−1	0	−1
McDoniel SO et al. (2010)	RCT	−1	−1	−1	−1	0	0	0	0
Webber KH et al. (2010)	RCT	−1	−1	−1	0	0	−1	0	−1
Werkman A et al. (2010)	RCT	0	−1	−2	−2	0	0	−2	−2
Harvey-Berino J et al. (2010)	RCT	0	−1	−1	−1	0	−1	0	0
Morgan PJ et al. (2011)	RCT	0	0	0	0	0	0	0	0
Collins CE et al. (2011)	RCT	−1	−1	−1	−1	0	−1	0	−1
Burke LE et al. (2011)	RCT	−1	−1	−1	−1	0	0	0	0
Gabriele JM et al. (2011)	RCT	0	0	−1	−1	0	−1	0	0
van Wier MF et al. (2011)	RCT	0	0	−1	−1	0	−1	−2	−1
Patrick K et al. (2011)	RCT	0	−1	−1	0	0	−1	0	0
Jebb SA et al. (2011)	RCT	0	0	−2	−2	0	0	0	−1
van Wier MF et al. (2012)	RCT	0	−1	−2	−1	0	0	0	−1
Anton SD et al. (2012)	RCT	0	0	0	0	−1	0	0	0
Brindal E et al. (2012)	RCT	−1	0	−1	−1	−1	−1	−2	−1
Morgan PJ et al. (2013)	RCT	0	0	0	0	0	0	0	0
Spring B et al. (2013)	RCT	0	−1	−1	−1	0	0	−2	−1
Gussenhoven AH et al. (2013)	RCT	−1	0	−2	−1	−1	−1	0	−1
Webber KH et al. (2013)	RCT	−1	−1	−1	0	0	−1	0	0
Turner-McGrievy GM et al. (2013)	RCT	−1	−1	−1	−1	0	−1	0	−1
Napolitano MA et al. (2013)	RCT	−1	−1	−1	−1	0	−1	0	−1
Brindal E et al. (2013)	RCT	0	0	−1	−1	0	0	0	0
Carter MC et al. (2013)	RCT	0	−2	−1	0	−1	0	0	−1
Imanaka M et al. (2013)	RCT	−1	−1	−1	−1	0	0	0	0
Collins CE et al. (2013)	RCT	−1	0	0	0	0	−1	−2	−1
Johnston CA et al. (2013)	RCT	0	−1	−1	−1	0	−1	0	0
Carnie A et al. (2013)	RCT	−1	−1	−1	−1	0	0	0	0
Allen JK et al. (2013)	RCT	−1	−1	−1	−1	0	−1	0	−1
Chung LM et al. (2014)	RCT	0	−1	0	0	0	−1	0	−1
Dennison L et al. (2014)	RCT	0	−2	−2	−1	0	0	0	−1
Morgan PJ et al. (2014)	RCT	0	0	0	0	−1	0	0	0
Barnes RD et al. (2014)	RCT	−1	−1	−1	0	0	−1	0	−1
Laing BY et al. (2014)	RCT	0	0	0	0	0	0	0	0
Okazaki et al. (2014)	Quasi-RCT	−1	−1	−1	−1	0	0	0	−1
Leahey TM et al. (2015)	RCT	−1	−1	−1	0	0	−1	0	0
Thomas JG et al. (2015)	RCT	0	0	−1	0	0	0	0	0
Azar KM et al. (2015)	RCT	−1	−1	−1	−1	0	−1	0	−1
Martin CK et al. (2015)	RCT	−1	−1	0	0	0	0	0	0
Fukuoka Y et al. (2015)	RCT	0	0	−1	−1	0	−1	−2	−1
Watson S et al. (2015)	RCT	0	0	0	0	0	0	−2	−1
Oh B et al. (2015)	RCT	0	−1	−2	−2	0	−1	−2	−1
Block G et al. (2015)	RCT	0	0	0	0	0	0	0	0
Unick JL et al. (2015)	RCT	−1	−1	−1	−1	0	−1	0	−1
Svetkey LP et al. (2015)	RCT	−1	−1	−1	−1	0	0	0	0
Crane MM et al. (2015)	RCT	0	−1	−2	−2	0	0	0	−1
Abdi J et al. (2015)	RCT	0	−1	−1	−1	−1	−1	0	−1
O'Brien T et al. (2016)	RCT	0	−1	−1	0	0	−1	0	−1
Godino JG et al. (2016)	RCT	0	0	−1	0	0	0	−2	0
Olson R et al. (2016)	RCT	−1	−1	−1	−1	−1	0	−2	−1
Little P et al. (2016)	RCT	0	0	−1	0	0	0	0	0
Hales S et al. (2016)	RCT	0	0	−1	−1	0	0	0	0
West DS et al. (2016)	RCT	0	−1	−1	−1	0	0	0	0
Jakicic JM et al. (2016)	RCT	0	0	0	0	0	0	0	0
Richardson CR et al. (2016)	RCT	−1	−1	−2	−2	0	0	−2	−1
Little P et al. (2017)	RCT	0	−1	−1	0	0	0	0	0
Naparstek J et al. (2017)	RCT	−1	−1	−1	−1	0	−1	−2	−1
Young AS et al. (2017)	RCT	−1	−1	−1	0	0	−1	0	−1
Gilmore LA et al. (2017)	RCT	−1	−1	−1	−1	0	0	0	0
Burke LE et al. (2017)	RCT	−1	−1	−1	−1	0	0	0	0
Spring B et al. (2017)	RCT	0	−1	−2	0	0	0	0	−1
Jane M et al. (2017)	RCT	0	−1	−1	−1	0	0	−2	−1
Phelan S et al. (2017)	RCT	−1	−1	−1	0	0	0	−2	−1
Petrella RJ et al. (2017)	RCT	0	0	−1	0	0	0	0	0
Thomas JG et al. (2017)	RCT	−1	−1	−1	0	0	0	0	0
Gomez-Marcos MA et al. (2018)	RCT	0	−1	0	0	0	0	0	0
Wang J et al. (2018)	RCT	0	−1	−1	−1	0	0	0	0
Jane M et al. (2018)	RCT	−1	−1	−1	−1	−2	0	−2	−1
Teeriniemi AM et al. (2018)	RCT	0	−1	−1	−1	0	0	0	0
Bender MS et al. (2018)	RCT	0	−1	−2	0	0	0	0	−1
Bender MS et al. (2018)	RCT	0	−1	−2	0	0	0	0	0
Alencar MK et al. (2019)	RCT	−1	−1	−1	−1	0	−1	0	−1
Ozaki I et al. (2019)	RCT	0	0	0	−2	0	0	0	0
Viglione C et al. (2019)	RCT	0	−1	−1	−1	0	0	−2	−1
Thomas JG et al. (2019)	RCT	0	0	−1	0	0	0	−2	−1
Patel ML et al. (2019)	RCT	0	−2	−2	0	0	0	0	−1
Apiñaniz A et al. (2019)	RCT	0	−1	−2	−1	0	0	0	−1
Kliemann N et al (2019)	RCT	0	−1	−1	−1	0	0	0	0
Dunn CG et al. (2019)	RCT	0	−1	−1	0	0	0	0	0
Forman EM et al. (2019)	RCT	0	0	0	−1	0	−1	−2	−1
Innes AQ et al. (2019)	RCT	0	−1	−2	−1	0	0	−2	−1
West DS et al. (2019)	RCT	0	−1	−1	−1	0	0	0	0
Lisón JF et al. (2020)	RCT	0	0	−1	0	0	0	0	0
West DS et al. (2020)	RCT	0	−1	−1	0	0	0	−2	−1
Hepdurgun C et al. (2020)	RCT	0	−1	−1	−1	0	−1	0	0

Supplementary Table S5. The categorized components of 97 articles.

Author(year)	1.1	1.2	1.3	1.4	1.5	1.6	1.7	2.2	2.3	2.4	2.7	3.1	3.3	4.1	4.3	5.1	6.2	10.1	10.2	10.4	10.8
Wylie-Rosett J et al. (2001)	√				√				√			√		√							
Tate DF et al. (2003)									√			√									
Womble LG et al. (2004)				√	√							√	√	√		√					
Rothert K et al. (2006)				√								√			√						
Adachi Y et al. (2007)	√							√	√												
Gold BC et al. (2007)	√		√					√	√	√		√		√							
McConnon A et al. (2007)	√							√	√	√						√					
Micco N et al. (2007)	√	√	√					√	√	√	√	√		√		√					
Webber Kh et al. (2008)												√		√							
Digenio AG et al. (2009)	√	√							√	√		√				√					
Morgan PJ et al. (2009)	√		√					√	√	√	√	√		√		√					
Turner-McGrievy GM et al. (2009)									√			√		√		√					
van Wier MF et al. (2009)	√								√	√	√	√				√					
Harvey-Berino J et al. (2010)								√				√		√		√					
McDoniel SO et al. (2010)				√					√	√											
Tanaka M et al. (2010)	√		√					√	√	√	√										
Webber KH et al. (2010)									√	√				√		√					
Werkman A et al. (2010)								√	√	√	√					√					
Burke LE et al. (2011)	√							√	√					√							
Collins CE et al. (2011)	√		√					√	√	√		√		√		√					
Gabriele JM et al. (2011)	√	√						√	√	√		√				√					
Jebb SA et al. (2011)			√						√	√		√		√							
Morgan PJ et al. (2011)	√		√					√	√	√	√	√									
Patrick K et al. (2011)	√							√	√			√				√					
van Wier MF et al. (2011)	√									√		√				√					
Anton SD et al. (2012)								√	√					√							
Brindal E et al. (2012)				√				√	√	√		√				√					
van Wier MF et al. (2012)	√								√			√				√					
Allen JK et al. (2013)	√							√		√											
Brindal E et al. (2013)	√								√	√						√				√	
Carnie A et al. (2013)				√										√		√					
Carter MC et al. (2013)	√		√					√	√												
Collins CE et al. (2013)	√							√	√	√		√				√					
Gussenhoven AH et al. (2013)									√					√		√					
Imanaka M et al. (2013)									√			√				√	√				
Johnston CA et al. (2013)				√					√	√		√		√							
Morgan PJ et al. (2013)	√		√					√	√	√		√									
Napolitano MA et al. (2013)	√		√						√	√		√				√					
Spring B et al. (2013)	√		√						√	√		√									
Turner-McGrievy GM et al. (2013)									√	√		√				√					
Webber KH et al. (2013)				√					√	√				√		√					√
Barnes RD et al. (2014)	√		√				√	√						√		√					
Chung LM et al. (2014)									√			√									
Dennison L et al. (2014)	√		√		√		√	√	√	√	√	√		√							
Laing BY et al. (2014)	√		√						√	√		√									
Morgan PJ et al. (2014)								√	√	√		√							√		
Okazaki H et al. (2014)	√		√	√								√				√					
Abdi J et al. (2015)	√											√				√		√			

Azar KM et al. (2015)										√		√					√				
Block G et al. (2015)	√		√			√			√	√	√	√				√			√		
Crane MM et al. (2015)								√	√	√		√						√			
Fukuoka Y et al. (2015)	√		√					√	√	√		√									
Leahey TM et al. (2015)								√	√	√	√			√		√		√			
Martin CK et al. (2015)									√	√	√					√					
Oh B et al. (2015)										√	√	√									
Svetkey LP et al. (2015)	√							√	√	√	√	√									
Thomas JG et al. (2015)	√		√			√		√	√	√	√	√									
Unick JL et al. (2015)		√						√	√	√	√					√					
Watson S et al. (2015)	√		√	√				√	√	√	√										
O'Brien T et al. (2016)								√	√	√		√		√							
Godino JG et al. (2016)	√				√			√				√									
Olson R et al. (2016)	√		√						√	√		√									√
Little P et al. (2016)			√							√		√		√		√					
Hales S et al. (2016)	√		√						√	√		√				√		√			
West DS et al. (2016)	√							√	√	√		√		√		√					
Jakicic JM et al. (2016)								√	√	√	√	√				√					
Richardson CR et al. (2016)	√	√						√	√			√									
Bender MS et al. (2017)	√		√						√	√		√				√					
Burke LE et al. (2017)	√							√	√	√											
Gilmore LA et al. (2017)	√							√	√	√						√					
Jane M et al. (2017)												√				√	√				
Little P et al. (2017)	√	√	√		√		√		√	√											
Naparstek J et al. (2017)	√	√						√	√	√	√					√					
Petrella RJ et al. (2017)	√								√	√		√		√		√		√			
Phelan S et al. (2017)	√		√					√	√	√		√				√					
Spring B et al. (2017)		√				√		√	√	√		√						√			
Thomas JG et al. (2017)	√							√	√	√	√	√		√							
Young AS et al. (2017)	√			√								√		√							
Bender MS et al. (2018)	√	√	√						√	√		√				√					
Gomez-Marcos MA et al. (2018)									√			√		√							
Jane M et al. (2018)				√								√				√					
Teeriniemi AM et al. (2018)		√						√	√	√				√		√					
Wang J et al. (2018)									√	√		√		√							
Alencar MK et al. (2019)		√						√				√				√					
Apiñaniz A et al. (2019)														√		√					
Dunn CG et al. (2019)									√		√			√							
Forman EM et al. (2019)				√					√					√							
Innes AQ et al. (2019)		√		√								√				√					
Kliemann N et al (2019)		√						√						√		√					
Ozaki I et al. (2019)	√		√						√	√		√				√				√	
Patel ML et al. (2019)	√	√	√						√	√				√							
Thomas JG et al. (2019)	√		√				√	√	√	√		√		√						√	
Viglione C et al. (2019)									√	√		√		√							
West DS et al. (2019)	√	√	√					√	√		√			√		√					
Hepdurgun C et al. (2020)								√	√			√				√					
Lisón JF et al. (2020)		√							√	√				√		√					
West DS et al. (2020)	√	√	√						√	√		√				√		√			
total	53	17	31	13	5	3	4	46	76	63	20	66	1	38	1	53	3	7	2	3	2
significantly different	17	7	13	2	1	2	1	14	24	21	6	24	0	5	1	16	2	2	1	1	1
not significantly different	15	4	7	3	3	0	3	16	18	15	6	10	1	10	0	13	0	0	0	2	0
not included in meta-analysis	21	6	11	8	1	1	0	16	34	27	8	32	0	23	0	24	1	5	1	0	1

Supplementary Table S6. The characteristics of the interventions retrieved in the meta-analysis.

Author (year)	Participants	Outcome	Group details
Wylie-Rosett et al. (2001) [13]	588 (Group 1, 97; Group 2, 183; Group 3, 194)	weight change	Group 1: Self-help through a workbook<br>Group 2: Workbook + network system<br>Group 3: Workbook + network system + staff consultation
Tate et al. (2003) [14]	92 (Basic, 46; E-counselling, 46)	weight change	Basic: Information through website<br>E-counselling: Basic + e-counseling via e-mail with assigned weight-loss counselor
Womble et al. (2004) [15]	47 (eDiets, 23; LEARN, 24)	weight change	eDiets: A commercial Internet weight-loss program in improving weight, cardiovascular health, and quality of life.<br>LEARN: A book that provided 16 step-by-step lessons for modifying eating, activity, and thinking habits.
Rothert et al. (2006) [16]	2862 (TES, 1475; IO, 1387)	weight change	TES: A tailored expert system<br>IO: Information-only Web-based weight management materials
Adachi et al. (2007) [17]	205 (KM, 46; K, 47; BM, 58; B, 54)	weight change	KM: KT* program with 6-month weight and targeted behavior's self-monitoring<br>K: KT program only<br>BM: An untailored self-help booklet with 7-month self-monitoring of weight and walking<br>B: The self-help booklet only<br>*The KT program consisted of twice-interactive letter communications including computer-tailored personal advice on treatment needs and behavioral modification.
Micco et al. (2007) [18]	123 (Internet-Only, 62; I+IPS, 61)	weight change	Internet-Only: The 12-month weight-loss program focused on changing eating and exercise habits through behavior modification techniques, which were taught through a series of online lessons.<br>I+IPS (Internet + in-person treatment): Participants in this condition had access to the same Internet treatment program

			previously described. However, they met once a month as a group in person.
Digenio AG et al. (2009) [19]	376 (HF-F2F, 74; LF-F2F, 76; HF-TEL, 76; HF-EMAIL, 74; SELF, 76)	weight change	HF-F2F: High-frequency face-to-face counseling<br>LF-F2F: Low-frequency face-to-face counseling<br>HF-TEL: High-frequency telephonic counseling<br>HF-EMAIL: High-frequency e-mail counseling<br>SELF: Self-help
Morgan et al. (2009) [20]	65 (Internet, 34; Information and self-help, 31)	weight change	Internet: Internet group participants used the study website to self-monitor diet and activity with feedback provided based on participants' online entries on seven occasions over 3 months + one face-to-face information session and a program booklet.<br>Information and self-help: One face-to-face information session and a program booklet.
Harvey-Berino et al. (2010) [21]	481 (In Person, 161; Hybrid, 158; Internet, 162)	weight change	In-person: Participants were weighed by study staff prior to beginning their group session. Each week, they received materials that covered the topic introduced that session. They were provided a commercially available calorie- and fat-counting book.<br>Hybrid: In-person + Internet<br>Internet: Participants in the internet condition met weekly in small groups of 15–20 in a secure online chat room. Participants had access to an online database to help monitor calorie intake.
McDoniel et al. (2010) [22]	132 (Usual Care, 56; SMART, 55)	weight change	Usual Care: The usual care group received a standard nutritional program in accordance with national guidelines.<br>SMART: Self-monitoring and resting metabolic rate technology
Tanaka et al. (2010) [23]	51 (KTP, 23; Control, 28)	weight change	KTP*: The KTP group read a booklet, set target behaviors, received advice, and self-monitored their weight and the targeted behaviors for 7 months.

			Control: The C group read the same booklet.<br>*The KTP (Kenkou-tatsujin) is a behavioral program assisted by computer-tailored advice.
Werkman et al. (2010) [12]	413 (Intervention, 203; Control, 197)	weight change	Intervention: Five program modules were provided to participants of the intervention group. Participants received computer-tailored feedback.<br>Control: During the total study period of two years, the control group was provided with newsletters with general information about the study.
Burke et al. (2011) [24]	210 (PR, 72; PDA, 68; PDA + FB, 70)	weight change	PR: Using a paper diary/record to self-monitoring<br>PDA: Using a personal digital assistant with dietary and exercise software<br>PDA + FB: PDA + daily feedback messages
Jebb et al. (2011) [25]	772 (Commercial program, 377; Standard care, 395)	weight change	Commercial program: 12 months of free membership to a commercial program<br>Standard care: 12 months of standard care as defined by national treatment guidelines
Brindal et al. (2012) [26]	8112 (Site I, 183; Site II, 3994; Site III, 3935)	weight change	Site I: Information-based<br>Site II: Site I + Include tools, such as a weight tracker, meal planner, and social networking platform.<br>Site III: Site II + Include a meal planner that offered recommendations that were personalized using an algorithm based on a user's preferences for certain foods.
van Wier et al. (2012) [27]	1386 (Phone, 462; Internet, 464; Control, 460)	weight change	Phone: Self-directed lifestyle brochures + phone counseling<br>Internet: Self-directed lifestyle brochures + e-mail counseling<br>Control: Self-directed lifestyle brochures
Allen et al. (2013) [28]	68 (IC, 18; IC + SP, 16; LIC + SP, 17; SP, 17)	weight change	IC: Intensive counseling intervention<br>IC + SP: Intensive counseling plus smartphone intervention<br>LIC + SP: A less intensive counseling plus smartphone intervention

			SP: Smartphone intervention only
Brindal et al. (2013) [29]	58 (Support, 28; Control, 30)	weight change	Support: Support app provided information, simplified food intake recording, rewarded positive behavior and prompted regular interaction through reminders and self-monitoring of weight and diet.<br>Control: Information available
Carter et al. (2013) [30]	128 (Smartphone, 43; Diary, 43; Website, 42)	weight change	Smartphone: Incorporate goal setting, self-monitoring of diet and activity, and feedback via weekly text message<br>Diary: Self-monitoring by paper diary<br>Website: Use an existing commercially available slimming website. The comparator groups delivered a similar self-monitoring intervention to the app, but by different modes of delivery.
Collins et al. (2013) [31]	301 (Basic, 143; Enhanced, 158)	weight change	Basic: Included self-efficacy, goal setting, self-monitoring, outcome expectations, and social support<br>Enhanced: Basic + personalized, system-generated enrollment reports; weekly automated system-generated, personalized e-feedback; an escalating reminder schedule
Morgan et al. (2013) [32]	159 (Online, 53; Resources, 54; Control, 52)	weight change	Online: Resources materials plus study website and e-feedback<br>Resources: Gender-tailored weight-loss materials (DVD, handbooks, pedometer, tape measure)<br>Control: Wait-list
Napolitano et al. (2013) [33]	52 (Facebook, 17; Facebook plus condition, 18; Waiting list, 17)	weight change	Facebook: Information; access to polls and healthy activity or eating event invitations<br>Facebook plus condition: Facebook + Receive additional theoretically driven intervention targets: goal setting, self-monitoring, and social support communicated via text messaging<br>Waiting list: Wait-list
Spring et al. (2013) [34]	70 (Standard, 35; +Mobile, 35)	weight change	Standard: Standard of care group treatment alone

			+Mobile: Standard + connective mobile technology system. Provide personal digital assistants (PDAs) to self-monitor diet and physical activity; provide biweekly coaching calls for 6 months.
Turner-McGrievy et al. (2013) [35]	85 (Mobile PA app, 48; No PA app used, 37)	weight change	Mobile PA app: Self-monitoring through mobile apps<br>No PA app used: Traditional self-monitoring
Barnes et al. (2014) [36]	89 (MIC, 30; NPC, 29; UC, 30)	weight change	MIC (motivational interviewing care): Utilize motivational interviewing (MI), supported by internet resources.<br>NPC (nutrition psychoeducation care): Designed as a five-session psychoeducation only, attention-control.<br>UC (usual care): Asked to continue working with their PC providers for weight-related concerns and not to start any structured/commercial weight-loss programs.
Dennison et al. (2014) [37]	786 (POWeR only, 264; POWeR plus coaching, 247; Waiting list, 275)	weight change	POWeR* only: A fully automated, tailored, Web-based weight management<br>POWeR plus coaching: POWeR + The coaching calls aimed to promote continued usage of the POWeR website and adherence to the recommendations within the website.<br>Waiting list: Wait-list<br>*The POWeR ("Positive Online Weight Reduction") web-based weight management consisted of weekly online sessions that emphasized self-monitoring, goal-setting, and cognitive/behavioral strategies.
Azar et al. (2015) [38]	64 (Intervention, 32; Control, 32)	weight change	Intervention: (1) 12 weekly classes, delivered through web-based, virtual small groups; (2) wireless and internet-connected (Wi-Fi) "smart" scales for weekly weighing.<br>Control: Wait list
Block et al. (2015) [39][a]	339 (Control, 176; Intervention, 163)	weight change	Intervention: Participants were provided tailored behavioral support for improvements: weekly emails suggested small-

			step goals and were linked to an individual Web page, along with a mobile phone app and automated phone calls.<br>Control: Usual-care
Crane et al. (2015) [40]	107 (REFIT, 53; Waitlist, 54)	weight change	REFIT*: The 6-month intervention was delivered via two face-to-face sessions followed by Internet contacts.<br>Waitlist: Wait-list<br>*REFIT (the Rethinking Eating and FITness) encouraged participants to create calorie deficits by making six 100-calorie changes to their daily intake while increasing physical activity, and the program encouraged customization through selection of specific diet strategies evaluated each week.
Fukuoka et al. (2015) [41]	61 (Control, 31; Intervention, 30)	weight change	Control: Control participants continued to receive standard medical care and attended the outcome assessment visits at 3 and 5 months but did not receive any intervention.<br>Intervention: Participants received the tailored diabetes prevention program through the run-in mobile app.
Martin et al. (2015) [42]	40 (SmartLoss, 20; Health Ed, 20)	weight change	SmartLoss: Participants were prescribed a 1200–1400 kcal/d diet and were provided with a smartphone, body weight scale, and accelerometer that wirelessly transmitted body weight and step data to a website.<br>Health Ed (Health Education): Receive health information via text messages or emails delivered to the smartphone during the study.
Oh et al. (2015) [43]	422 (Intervention, 212; Control, 210)	weight change	Intervention*: Provided with remote monitoring and health care services in addition to the existing treatment.<br>Control*: The control group was provided with only the existing treatment.<br>*Pedometers were given to all of the patients.

Watson et al. (2015) [44]	65 (Intervention, 32; Control, 33)	weight change	Intervention: Provided with the Web-based program, which supported positive dietary and physical activity changes and assisted in managing weight.<br>Control: Continued with their usual self-care
Jakicic et al. (2016) [45]	470 (SBWI, 233; EWLI, 237)	weight change	SBWI: Standard behavioral weight-loss intervention<br>EWLI: Technology-enhanced weight-loss intervention
Olson et al. (2016) [46]	472 (Control, 225; Intervention, 247)	weight change	Intervention: Intervention activities were facilitated through a mobile-friendly Web site.<br>Control: Wait list
Richardson et al. (2016) [47]	255 (Time-Based, 85; Simple Pedometer, 86; Enhanced Pedometer, 84)	weight change	Time-based: Time-based walking goals<br>Simple Pedometer: Simple pedometer-based walking goals<br>Enhanced Pedometer: Web-enhanced pedometer feedback goals with internet-mediated feedback
West et al. (2016) [48]	398 (BT, 199; BT + MI, 199)	weight change	BT: A 36-session group internet behavioral weight control treatment<br>BT + MI (motivational interviewing): BT + 6 individual MI chat sessions
Bender et al. (2017) [49]	45 (Intervention, 22; Active Waitlist, 23)	weight change	Intervention: Intervention participants were trained on using the Fitbit accelerometer to self-monitor. They joined the study’s private Facebook group for virtual social support, coaching, and weekly education topics posted by research staff. Participants were encouraged to join the Facebook discussions at least once a week.<br>Active waitlist: Wait list
Gilmore et al. (2017) [50]	40 (WIC Moms, 20; E-Moms, 20)	weight change	WIC* Moms: Usual care<br>E-Moms: A personalized health intervention delivered via a smartphone<br>*WIC: postpartum women, infants, and children service
Jane et al. (2017) [51]	137 (Control, 45; Pamphlet, 46; Facebook, 46)	weight change	Control: Standard care<br>Pamphlet: Received the same program as the Facebook group in a booklet<br>Facebook: Received the program

			within a Facebook group, along with a support network
Naparstek et al. (2017) [52]	125 (IBWL, 83; Control, 42)	weight change	IBWL (Internet behavioral weight loss): An online platform where participants received weight loss, calorie, and physical activity goals; 12 weekly multimedia lessons focused on behavioral weight-loss strategies; and a self-monitoring platform where participants reported their weight, calorie, and activity information and received automated, tailored feedback each week.<br>Control: As usual
Phelan et al. (2017) [53]	371 (Standard care, 197; Intervention program, 174)	weight change	Standard care: Standard care<br>Intervention program: Standard care + A 12-month primarily internet-based weight-loss program
Spring et al. (2017) [54]	96 (Standard, 32; Technology Supported, 32; Self-Guided, 32)	weight change	Standard: Attended weekly 90-minute group sessions led by a psychologist or exercise physiologist and focused on nutrition, MVPA (moderate to vigorous physical activity), and behavior change strategies. A 30-minute guided walking exercise was offered after group sessions. Standard and self-guided participants received the same calorie counting book and paper diaries.<br>Technology supported: Standard + An Android smartphone with study-designed ENGAGED* app and accelerometer for 6 months.<br>Self-guided: Attend one 60-minute group session at which treatment assignment was revealed and participants received their weight-loss target, a calorie and fat gram counting book, and 6 months of daily paper self-monitoring diaries.<br>*ENGAGED: E-Networks Guiding Adherence to Goals in Exercise and Diet
Bender et al. (2018) [55]	67 (Intervention, 33; Waitlist Control, 34)	weight change	Intervention: Participants 1) attended 5 in-person intervention office visits, 2) tracked real-time steps by

			wearing a Fitbit Zip on their torso at least 10 h/day, 3) logged daily food/drink intake and weekly home weights on a mobile app/diary, and 4) received weekly postings of discussion topics (related to weight loss, PA, and healthy eating) on the study's private Facebook group site to reinforce healthy behaviors learned during in-person intervention sessions.<br>Waitlist control: Wait list
Teeriniemi AM et al. (2018) [56]	532 (CBT, 85; CBT + HBCSS, 88; SHG, 87; SHG + HBCSS, 92; Control, 89; Control + HBCSS, 91)	weight change	CBT*1: Eight sessions led by a nutritionist<br>CBT + HBCSS*2: CBT + HBCSS<br>SHG: Self-help guidance-based group counselling (two sessions led by a nurse)<br>SHG + HBCSS: SHG + HBCSS<br>Control: As usual<br>Control + HBCSS: Control + HBCSS<br>*1CBT: methods of cognitive behavioral therapy<br>*2HBCSS: Information and communication technology health behavior change support system
Alencar et al. (2019) [57]	25 (VC, 13; CON, 12)	weight change	VC: The video conferencing group with health coaching; mobile app<br>CON: The participants received the Bluetooth, scale, watch, and blood pressure cuff but did not have weekly health coaching sessions.
Kliemann et al. (2019) [58]	81 (Top Tips Only, 28; Top Tips plus, 27; Waiting list, 26)	weight change	Top Tips Only: The Ten Top Tips is an intervention based on the habit formation theory that promotes a set of weight management behaviors alongside advice about repetition in a consistent context.<br>Top Tips plus: An additional tip targeting self-regulatory strategies to resist tempting food<br>Waiting list: Wait list
Ozaki et al. (2019) [59]	80 (ESG, 25; SSG, 27; CG, 28)	weight change	ESG (Enhanced Support Group): SSG + Four remote support sessions based on Supportive Accountability in addition to the

			SSG<br>SSG (Standard Support Group): Provide support via website and two face-to-face group guidance sessions, at the beginning and at the end of the program along with monthly general emails throughout the program<br>CG: Wait-list
Thomas et al. (2019) [60]	276 (SMART, 114; GROUP, 106; CONTROL, 56)	weight change	SMART (A primarily smartphone-based behavioral obesity treatment): Treated with online lessons, self-monitoring, and feedback and monthly weigh-ins<br>GROUP (A more intensive group-based behavioral obesity treatment): Treated with meetings weekly for 6 months, bi-weekly for 6 months, and monthly for 6-months and self-monitoring via paper diaries with written feedback<br>CONTROL (A control condition): Self-monitoring via paper diaries with written feedback and monthly weigh-ins
Hepdurgun C et al. (2020) [61]	101 (IBT, 51; EE, 50)	weight change	IBT (internet behavior therapy): The participants in the IBT group were provided access to an Internet program that provided videos teaching behavioral weight-loss skills as well as a self-monitoring platform to calculate the daily calorie balance.<br>EE (e-mail education): The participants in the EE group received weekly e-mails with information on healthy eating, physical exercises, and weight loss for 8 weeks.
Lisón et al. (2020) [62]	105 (Internet-Based, 55; Wait-list, 50)	BMI change	Internet-based: Participants received a 3-month multimedia, interactive, and self-administered online intervention program comprised of nine modules.<br>Waitlist: Wait-list

[a]The SD of the weight loss data in this study was incorrect; thus, we contacted the author and received the original data.

Study or Subgroup	web-based intervention Mean	SD	Total	Control Mean	SD	Total	Weight	Std. Mean Difference IV, Random, 95% CI	Year	A	B	C	D	E	F	G
Wylie-Rosett J, 2001	−4.7	1	183	−2.2	1.3	97	3.6%	−2.24 [−2.55, −1.93]	2001	?	?	?	?	+	?	+
Womble LG, 2004	−0.8	3.6	23	−3.3	4.1	24	3.0%	0.64 [0.05, 1.22]	2004	?	?	?	?	+	?	+
Adachi Y, 2007	−2.2	3	44	−1.4	2.4	50	3.5%	−0.29 [−0.70, 0.11]	2007	?	?	?	?	+	?	+
Morgan PJ, 2009	−4.8	4.5	34	−3	4.5	31	3.3%	−0.40 [−0.89, 0.10]	2009	+	+	+	+	+	+	+
Harvey-Berino J, 2010	−5.5	5.6	159	−8	6.1	150	3.8%	0.43 [0.20, 0.65]	2010	+	?	?	?	+	?	+
Tanaka M, 2010	−2.4	3.2	23	−1.6	2.8	28	3.1%	−0.26 [−0.82, 0.29]	2010	?	?	?	?	+	?	+
McDoniel SO, 2010	−3.5	4.3	55	−3.7	4.2	56	3.5%	0.05 [−0.33, 0.42]	2010	?	?	?	?	+	+	+
Werkman A, 2010	−1.9	3.1	166	−1.6	3	169	3.8%	−0.10 [−0.31, 0.12]	2010	+	?	−	−	+	+	−
Burke LE, 2011	−5.5	7	68	−5.3	5.9	62	3.6%	−0.03 [−0.37, 0.31]	2011	?	?	?	?	+	+	+
Jebb SA, 2011	−6.7	6.5	230	−3.2	4.8	214	3.8%	−0.61 [−0.80, −0.42]	2011	+	+	−	−	+	+	+
van Wier MF, 2012	−1.9	5.7	450	−1.1	7	448	3.9%	−0.13 [−0.26, 0.01]	2012	+	+	?	?	+	?	−
Brindal E, 2012	−4.2	4.3	213	−4.2	4.3	16	3.2%	0.00 [−0.51, 0.51]	2012	?	+	?	?	?	?	−
Morgan PJ, 2013	−5.8	1.2	33	−0.6	3.1	37	3.0%	−2.14 [−2.74, −1.55]	2013	+	+	+	+	?	+	+
Barnes RD, 2014	−1.5	3	28	−0.2	2.8	29	3.2%	−0.44 [−0.97, 0.08]	2014	?	?	?	+	+	?	+
Dennison L, 2014	−2.9	4.4	39	−0.4	2.4	158	3.5%	−0.86 [−1.22, −0.50]	2014	+	−	−	?	+	+	+
Azar KM, 2015	−3.6	4	32	−0.4	2	32	3.2%	−1.00 [−1.52, −0.48]	2015	?	?	?	?	+	?	+
Crane MM, 2015	−4.9	3.3	53	−0.6	3.4	54	3.4%	−1.27 [−1.69, −0.86]	2015	+	?	−	−	+	+	+
Watson S, 2015	−4.4	3.6	25	−0.5	3.1	32	3.1%	−1.16 [−1.72, −0.59]	2015	+	+	+	+	+	+	−
Jakicic JM, 2016	−3.5	7.1	237	−5.9	7	233	3.8%	0.34 [0.16, 0.52]	2016	+	+	+	+	+	+	?
Olson R, 2016	−2.4	6.7	229	1	5.7	223	3.8%	−0.55 [−0.73, −0.36]	2016	?	?	?	?	?	+	−
Richardson CR, 2016	−1.9	3.7	84	−0.6	3.8	86	3.7%	−0.35 [−0.65, −0.04]	2016	?	?	−	−	+	+	−
Phelan S, 2017	−3.2	5.4	174	−0.9	5.7	193	3.8%	−0.41 [−0.62, −0.21]	2017	?	?	?	+	+	+	−
Jane M, 2017	−3.5	3.8	22	−1.8	2.1	18	2.9%	−0.53 [−1.16, 0.11]	2017	?	?	?	?	−	+	−
Naparstek J, 2017	−4.1	4.4	42	−1.6	4.4	83	3.5%	−0.56 [−0.94, −0.19]	2017	?	?	?	?	+	?	−
Teeriniemi AM, 2018	−1.4	5	79	−0.4	4.6	68	3.6%	−0.21 [−0.53, 0.12]	2018	+	?	?	?	+	+	+
Bender MS, 2018	−1.1	1.2	33	−0.3	0.8	34	3.2%	−0.78 [−1.28, −0.28]	2018	+	?	−	+	+	+	+
Kliemann N, 2019	−4.5	10	17	−0.2	13.3	26	3.0%	−0.35 [−0.96, 0.27]	2019	+	?	?	?	+	+	+
Hepdurgun C, 2020	−2.3	2.1	51	−0.7	1.6	50	3.5%	−0.85 [−1.26, −0.44]	2020	+	?	?	?	+	?	+
Lisón JF, 2020	−1.1	3	55	0.3	2.9	50	3.5%	−0.47 [−0.86, −0.08]	2020	+	?	?	+	+	+	−
**Total (95% CI)**			**2881**			**2751**	**100.0%**	**−0.49 [−0.70, −0.28]**								

Heterogeneity: $Tau^2 = 0.30$; $Chi^2 = 379.17$, df = 28 ($P < 0.00001$); $I^2 = 93\%$

Test for overall effect: Z = 4.51 ($P < 0.00001$)

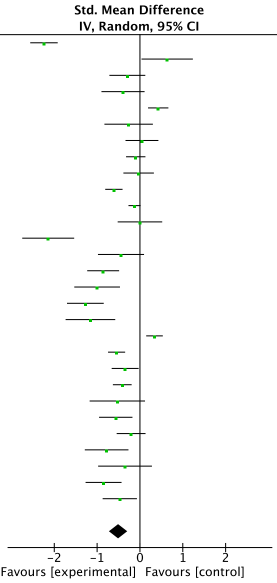

Risk of bias legend

(**A**) Random sequence generation (selection bias)
(**B**) Allocation concealment (selection bias)
(**C**) Blinding of participants and personnel (performance bias)
(**D**) Blinding of outcome assessment (detection bias)
(**E**) Incomplete outcome data (attrition bias)
(**F**) Selective reporting (reporting bias)
(**G**) Other bias

Supplementary Figure S1. Meta-analysis for mean weight change comparing web-based interventions with offline control groups through computers. The forest plot shows standardized mean differences (SMD) for each article with 95% confidence intervals (CI). The diamond at the bottom of the graph means the meta-analyzed measure of effect and the lateral points of which indicate CIs for this estimate. A positive value reflects web-based interventions are more effective for weight loss than offline interventions. The area of each green square is proportional to the study's weight in the meta-analysis.

Study or Subgroup	web-based intervention Mean	SD	Total	Control Mean	SD	Total	Weight	Std. Mean Difference IV, Random, 95% CI	Year
Allen JK, 2013	−5.4	4	16	−2.5	4.1	18	6.0%	−0.70 [−1.39, −0.00]	2013
Carter MC, 2013	−4.6	5.4	43	−2.9	6	43	7.4%	−0.30 [−0.72, 0.13]	2013
Spring B, 2013	−4.5	6.5	29	−1	4.4	28	6.8%	−0.62 [−1.15, −0.09]	2013
Turner−McGrievy GM, 2013	−3.7	10.1	48	−0.5	9.1	37	7.4%	−0.33 [−0.76, 0.10]	2013
Napolitano MA, 2013	−0.6	2.4	17	−0.2	2.6	17	6.1%	−0.16 [−0.83, 0.52]	2013
Brindal E, 2013	−3.2	1.7	21	−2.2	1.8	23	6.5%	−0.56 [−1.16, 0.04]	2013
Fukuoka Y, 2015	−6.2	5.9	28	0.3	2.7	28	6.5%	−1.40 [−1.99, −0.81]	2015
Oh B, 2015	−2.3	3.6	181	−0.9	2.8	153	8.2%	−0.43 [−0.65, −0.21]	2015
Block G, 2015	−3.1	3.6	136	−1.1	2.8	156	8.2%	−0.62 [−0.86, −0.39]	2015
Martin CK, 2015	−7.8	2	19	−0.6	2	19	4.3%	−3.52 [−4.58, −2.47]	2015
Bender MS, 2017	−2.9	2.4	22	−0.2	2	23	6.3%	−1.20 [−1.84, −0.56]	2017
Gilmore LA, 2017	−0.1	0.9	19	1.8	0.9	16	5.3%	−2.06 [−2.90, −1.22]	2017
Spring B, 2017	−4.7	6.4	32	−6.6	6.4	32	7.0%	0.29 [−0.20, 0.79]	2017
Ozaki I, 2019	−3.7	2.8	24	0.6	2.9	22	6.2%	−1.48 [−2.14, −0.82]	2019
Thomas JG, 2019	−5.5	8.7	114	−6.4	10.3	56	7.9%	0.10 [−0.22, 0.42]	2019
**Total (95% CI)**			**749**			**671**	**100.0%**	**−0.75 [−1.06, −0.44]**	

Heterogeneity: $Tau^2 = 0.29$; $Chi^2 = 94.97$, df = 14 ($P < 0.00001$); $I^2 = 85\%$
Test for overall effect: $Z = 4.77$ ($P < 0.00001$)

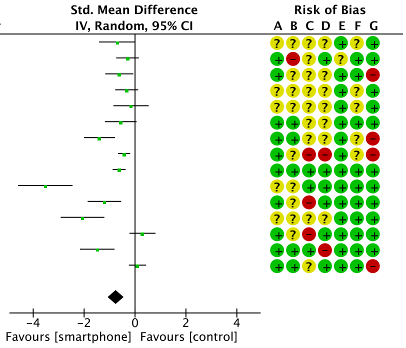

Risk of bias legend
(**A**) Random sequence generation (selection bias)
(**B**) Allocation concealment (selection bias)
(**C**) Blinding of participants and personnel (performance bias)
(**D**) Blinding of outcome assessment (detection bias)
(**E**) Incomplete outcome data (attrition bias)
(**F**) Selective reporting (reporting bias)
(**G**) Other bias

Supplementary Figure S2. Meta-analysis for mean weight change comparing web-based interventions with offline control groups through smartphone apps. The forest plot shows standardized mean differences (SMD) for each article with 95% confidence intervals (CI). The diamond at the bottom of the graph means the meta-analyzed measure of effect and the lateral points of which indicate CIs for this estimate. A positive value reflects web-based interventions are more effective for weight loss than offline interventions. The area of each green square is proportional to the study's weight in the meta-analysis.

Study or Subgroup	web-based intervention Mean	SD	Total	Control Mean	SD	Total	Weight	Std. Mean Difference IV, Random, 95% CI	Year
Wylie-Rosett J, 2001	−4.7	1	183	−2.2	1.3	97	12.8%	−2.24 [−2.55, −1.93]	2001
Womble LG, 2004	−0.8	3.6	23	−3.3	4.1	24	11.9%	0.64 [0.05, 1.22]	2004
Adachi Y, 2007	−2.2	3	44	−1.4	2.4	50	12.5%	−0.29 [−0.70, 0.11]	2007
Morgan PJ, 2009	−4.8	4.5	34	−3	4.5	31	12.2%	−0.40 [−0.89, 0.10]	2009
Tanaka M, 2010	−2.4	3.2	23	−1.6	2.8	28	12.0%	−0.26 [−0.82, 0.29]	2010
Harvey-Berino J, 2010	−5.5	5.6	159	−8	6.1	150	12.9%	0.43 [0.20, 0.65]	2010
McDoniel SO, 2010	−3.5	4.3	55	−3.7	4.2	56	12.6%	0.05 [−0.33, 0.42]	2010
Werkman A, 2010	−1.9	3.1	166	−1.6	3	169	13.0%	−0.10 [−0.31, 0.12]	2010
**Total (95% CI)**			**687**			**605**	**100.0%**	**−0.28 [−0.94, 0.38]**	

Heterogeneity: $Tau^2 = 0.86$; $Chi^2 = 207.36$, df = 7 ($P < 0.00001$); $I^2 = 97\%$
Test for overall effect: Z = 0.83 (P = 0.41)

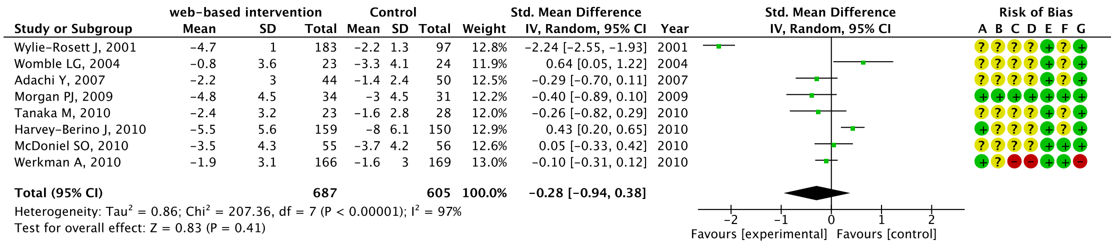

<u>Risk of bias legend</u>
(**A**) Random sequence generation (selection bias)
(**B**) Allocation concealment (selection bias)
(**C**) Blinding of participants and personnel (performance bias)
(**D**) Blinding of outcome assessment (detection bias)
(**E**) Incomplete outcome data (attrition bias)
(**F**) Selective reporting (reporting bias)
(**G**) Other bias

Supplementary Figure S3. Meta-analysis for mean weight change comparing web-based interventions with offline control groups from papers published from 2001 to 2010. The forest plot shows standardized mean differences (SMD) for each article with 95% confidence intervals (CI). The diamond at the bottom of the graph means the meta-analyzed measure of effect and the lateral points of which indicate CIs for this estimate. A positive value reflects web-based interventions are more effective for weight loss than offline interventions. The area of each green square is proportional to the study's weight in the meta-analysis.

Study or Subgroup	web-based intervention Mean	SD	Total	Control Mean	SD	Total	Weight	Std. Mean Difference IV, Random, 95% CI	Year	A	B	C	D	E	F	G
Burke LE, 2011	−5.5	7	68	−5.3	5.9	72	5.7%	−0.03 [−0.36, 0.30]	2011	?	?	?	?	+	+	+
Jebb SA, 2011	−6.7	6.5	230	−3.3	4.8	214	6.2%	−0.59 [−0.78, −0.40]	2011	+	+	−	−	+	+	+
van Wier MF, 2012	−1.9	5.7	450	−1.1	7	448	6.4%	−0.13 [−0.26, 0.01]	2012	+	+	?	?	+	?	−
Brindal E, 2012	−4.2	4.3	213	−4.2	4.3	16	4.9%	0.00 [−0.51, 0.51]	2012	?	+	?	?	?	?	−
Carter MC, 2013	−4.6	5.4	43	−2.9	6	43	5.3%	−0.30 [−0.72, 0.13]	2013	+	−	?	+	?	+	+
Morgan PJ, 2013	−5.8	1.2	33	−0.6	3.1	37	4.5%	−2.14 [−2.74, −1.55]	2013	+	+	+	+	?	+	+
Napolitano MA, 2013	−0.6	2.4	17	−0.2	2.6	17	4.1%	−0.16 [−0.83, 0.52]	2013	?	?	?	?	+	?	+
Spring B, 2013	−4.5	6.5	29	−1	4.4	28	4.8%	−0.62 [−1.15, −0.09]	2013	+	?	?	?	+	+	−
Turner−McGrievy GM, 2013	−3.7	10.4	48	−0.5	9.1	37	5.3%	−0.32 [−0.75, 0.11]	2013	?	?	?	?	+	?	+
Allen JK, 2013	−5.4	4	16	−2.5	4.1	18	4.0%	−0.70 [−1.39, −0.00]	2013	?	?	?	?	+	?	+
Brindal E, 2013	−3.2	1.7	21	−2.2	1.8	23	4.4%	−0.56 [−1.16, 0.04]	2013	+	+	?	?	+	+	+
Barnes RD, 2014	−1.5	3	28	−0.2	2.8	29	4.8%	−0.44 [−0.97, 0.08]	2014	?	?	?	+	+	?	+
Dennison L, 2014	−2.9	4.4	39	−0.4	2.4	158	5.6%	−0.86 [−1.22, −0.50]	2014	+	−	−	?	+	+	+
Crane MM, 2015	−4.9	3.3	53	−0.6	3.4	54	5.3%	−1.27 [−1.69, −0.86]	2015	+	?	−	−	+	+	+
Fukuoka Y, 2015	−6.2	5.9	28	0.3	2.7	28	4.5%	−1.40 [−1.99, −0.81]	2015	+	+	?	?	+	?	−
Martin CK, 2015	−7.8	2	19	−0.6	2	19	2.7%	−3.52 [−4.58, −2.47]	2015	?	?	+	+	+	+	+
Block G, 2015	−3.1	3.6	136	−1.1	2.8	156	6.1%	−0.62 [−0.86, −0.39]	2015	+	+	+	+	+	+	+
Oh B, 2015	−2.3	3.6	181	−0.9	2.8	153	6.1%	−0.43 [−0.65, −0.21]	2015	+	?	−	−	+	?	−
Watson S, 2015	−4.4	3.6	25	−0.5	3.1	32	4.6%	−1.16 [−1.72, −0.59]	2015	+	+	+	+	+	+	−
Azar KM, 2015	−3.6	4	32	−0.4	2	32	4.8%	−1.00 [−1.52, −0.48]	2015	?	?	?	?	+	?	+
**Total (95% CI)**			**1709**			**1614**	**100.0%**	**−0.72 [−0.95, −0.50]**								

Heterogeneity: $Tau^2 = 0.20$; $Chi^2 = 143.39$, df = 19 ($P < 0.00001$); $I^2 = 87\%$
Test for overall effect: Z = 6.33 ($P < 0.00001$)

Std. Mean Difference
IV, Random, 95% CI

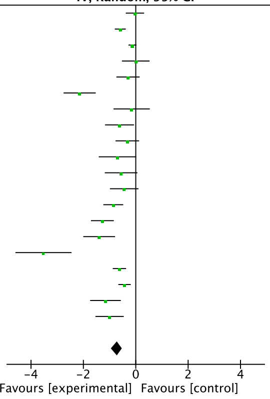

Risk of bias legend
(**A**) Random sequence generation (selection bias)
(**B**) Allocation concealment (selection bias)
(**C**) Blinding of participants and personnel (performance bias)
(**D**) Blinding of outcome assessment (detection bias)
(**E**) Incomplete outcome data (attrition bias)
(**F**) Selective reporting (reporting bias)
(**G**) Other bias

Supplementary Figure S4. Meta-analysis for mean weight change comparing web-based interventions with offline control groups from papers published from 2011 to 2015. The forest plot shows standardized mean differences (SMD) for each article with 95% confidence intervals (CI). The diamond at the bottom of the graph means the meta-analyzed measure of effect and the lateral points of which indicate CIs for this estimate. A positive value reflects web-based interventions are more effective for weight loss than offline interventions. The area of each green square is proportional to the study's weight in the meta-analysis.

Study or Subgroup	web-based intervention Mean	SD	Total	Control Mean	SD	Total	Weight	Std. Mean Difference IV, Random, 95% CI	Year	A	B	C	D	E	F	G
Jakicic JM, 2016	−3.5	7.1	237	−5.9	7	233	7.6%	0.34 [0.16, 0.52]	2016	+	+	+	+	+	+	?
Olson R, 2016	−2.4	6.7	229	1	5.7	223	7.5%	−0.55 [−0.73, −0.36]	2016	?	?	?	?	?	+	−
Richardson CR, 2016	−1.9	3.7	84	−0.6	3.8	86	7.0%	−0.35 [−0.65, −0.04]	2016	?	?	−	−	+	+	−
Jane M, 2017	−3.5	3.8	22	−1.8	2.1	18	5.2%	−0.53 [−1.16, 0.11]	2017	?	?	?	?	−	+	−
Naparstek J, 2017	−4.1	4.4	42	−1.6	4.4	83	6.7%	−0.56 [−0.94, −0.19]	2017	?	?	?	?	+	?	−
Phelan S, 2017	−3.2	5.4	174	−0.9	5.7	193	7.5%	−0.41 [−0.62, −0.21]	2017	?	?	?	+	+	+	−
Spring B, 2017	−4.7	6.4	32	−6.6	6.4	32	6.0%	0.29 [−0.20, 0.79]	2017	+	?	−	+	+	+	+
Bender MS, 2017	−2.9	2.4	22	−0.3	2	23	5.2%	−1.16 [−1.79, −0.52]	2017	+	?	−	+	+	+	+
Gilmore LA, 2017	−0.1	0.9	19	1.8	0.9	16	4.1%	−2.06 [−2.90, −1.22]	2017	?	?	?	?	+	+	+
Bender MS, 2018	−1.1	1.2	33	−0.3	0.8	34	6.0%	−0.78 [−1.28, −0.28]	2018	+	?	−	+	+	+	+
Teeriniemi AM, 2018	−1.4	5	79	−0.4	4.6	68	6.9%	−0.21 [−0.53, 0.12]	2018	+	?	?	?	+	+	+
Ozaki I, 2019	−3.7	2.8	24	0.6	2.9	22	5.0%	−1.48 [−2.14, −0.82]	2019	+	+	+	−	+	+	+
Thomas JG, 2019	−5.5	8.7	114	−6.4	10.3	56	7.0%	0.10 [−0.22, 0.42]	2019	+	+	?	+	+	+	−
Kliemann N, 2019	−4.5	10	17	−0.2	13.3	26	5.3%	−0.35 [−0.96, 0.27]	2019	+	?	?	?	+	+	+
Hepdurgun C, 2020	−2.3	2.1	51	−0.7	1.6	50	6.5%	−0.85 [−1.26, −0.44]	2020	+	?	?	?	+	?	+
Lisón JF, 2020	−1.1	3	55	0.3	2.9	50	6.6%	−0.47 [−0.86, −0.08]	2020	+	?	?	+	+	+	−
**Total (95% CI)**			**1234**			**1213**	**100.0%**	**−0.50 [−0.74, −0.25]**								

Heterogeneity: $Tau^2 = 0.20$; $Chi^2 = 116.92$, df = 15 ($P < 0.00001$); $I^2 = 87\%$
Test for overall effect: $Z = 3.95$ ($P < 0.0001$)

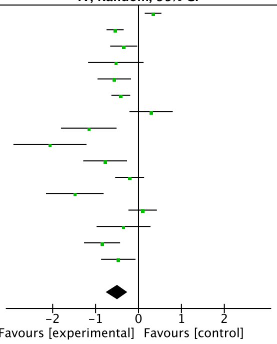

Risk of bias legend
(**A**) Random sequence generation (selection bias)
(**B**) Allocation concealment (selection bias)
(**C**) Blinding of participants and personnel (performance bias)
(**D**) Blinding of outcome assessment (detection bias)
(**E**) Incomplete outcome data (attrition bias)
(**F**) Selective reporting (reporting bias)
(**G**) Other bias

Supplementary Figure S5. Meta-analysis for mean weight change comparing web-based interventions with offline control groups from papers published from 2016 to 2020. The forest plot shows standardized mean differences (SMD) for each article with 95% confidence intervals (CI). The diamond at the bottom of the graph means the meta-analyzed measure of effect and the lateral points of which indicate CIs for this estimate. A positive value reflects web-based interventions are more effective for weight loss than offline interventions. The area of each green square is proportional to the study's weight in the meta-analysis.

Study or Subgroup	web-based intervention Mean	SD	Total	Control Mean	SD	Total	Weight	Std. Mean Difference IV, Random, 95% CI	Year
Napolitano MA, 2013	−0.6	2.4	17	−0.2	2.6	17	14.2%	−0.16 [−0.83, 0.52]	2013
Brindal E, 2013	−3.2	1.7	21	−2.2	1.8	23	17.1%	−0.56 [−1.16, 0.04]	2013
Dennison L, 2014	−2.9	4.4	39	−0.4	2.4	158	37.2%	−0.86 [−1.22, −0.50]	2014
Hepdurgun C, 2020	−2.3	2.1	51	−0.7	1.6	50	31.5%	−0.85 [−1.26, −0.44]	2020
**Total (95% CI)**			**128**			**248**	**100.0%**	**−0.70 [−0.98, −0.43]**	

Heterogeneity: $Tau^2 = 0.02$; $Chi^2 = 3.91$, df = 3 (P = 0.27); $I^2 = 23\%$
Test for overall effect: Z = 5.05 (P < 0.00001)

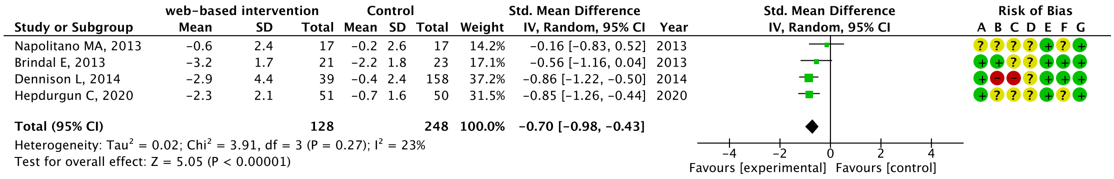

Risk of bias legend
(**A**) Random sequence generation (selection bias)
(**B**) Allocation concealment (selection bias)
(**C**) Blinding of participants and personnel (performance bias)
(**D**) Blinding of outcome assessment (detection bias)
(**E**) Incomplete outcome data (attrition bias)
(**F**) Selective reporting (reporting bias)
(**G**) Other bias

Supplementary Figure S6. Meta-analysis for mean weight change comparing web-based interventions with offline control groups for <3 months of duration. The forest plot shows standardized mean differences (SMD) for each article with 95% confidence intervals (CI). The diamond at the bottom of the graph means the meta-analyzed measure of effect and the lateral points of which indicate CIs for this estimate. A positive value reflects web-based interventions are more effective for weight loss than offline interventions. The area of each green square is proportional to the study's weight in the meta-analysis.

Study or Subgroup	web-based intervention Mean	SD	Total	Control Mean	SD	Total	Weight	Std. Mean Difference IV, Random, 95% CI	Year
Morgan PJ, 2009	−4.8	4.5	34	−3	4.5	31	3.7%	−0.40 [−0.89, 0.10]	2009
McDoniel SO, 2010	−3.5	4.3	55	−3.7	4.2	56	4.0%	0.05 [−0.33, 0.42]	2010
Harvey–Berino J, 2010	−5.5	5.6	159	−8	6.1	150	4.4%	0.43 [0.20, 0.65]	2010
Burke LE, 2011	−5.5	7	68	−5.3	5.9	72	4.1%	−0.03 [−0.36, 0.30]	2011
Brindal E, 2012	−4.2	4.3	213	−4.2	4.3	16	3.7%	0.00 [−0.51, 0.51]	2012
Allen JK, 2013	−5.4	4	16	−2.5	4.1	18	3.1%	−0.70 [−1.39, −0.00]	2013
Carter MC, 2013	−4.6	5.4	43	−2.9	6	43	3.9%	−0.30 [−0.72, 0.13]	2013
Morgan PJ, 2013	−5.8	1.2	33	−0.6	3.1	37	3.4%	−2.14 [−2.74, −1.55]	2013
Turner–McGrievy GM, 2013	−3.7	10.4	48	−0.5	9.1	37	3.9%	−0.32 [−0.75, 0.11]	2013
Barnes RD, 2014	−1.5	3	28	−0.2	2.8	29	3.6%	−0.44 [−0.97, 0.08]	2014
Martin CK, 2015	−7.8	2	19	−0.6	2	19	2.2%	−3.52 [−4.58, −2.47]	2015
Azar KM, 2015	−3.6	4	32	−0.4	2	32	3.6%	−1.00 [−1.52, −0.48]	2015
Block G, 2015	−3.1	3.6	136	−1.1	2.8	156	4.3%	−0.62 [−0.86, −0.39]	2015
Crane MM, 2015	−4.9	3.3	53	−0.6	3.4	54	3.9%	−1.27 [−1.69, −0.86]	2015
Fukuoka Y, 2015	−6.2	5.9	28	0.3	2.7	28	3.4%	−1.40 [−1.99, −0.81]	2015
Oh B, 2015	−2.3	3.6	181	−0.9	2.8	153	4.4%	−0.43 [−0.65, −0.21]	2015
Olson R, 2016	−2.4	6.7	229	1	5.7	223	4.4%	−0.55 [−0.73, −0.36]	2016
Richardson CR, 2016	−1.9	3.7	84	−0.6	3.8	86	4.2%	−0.35 [−0.65, −0.04]	2016
Naparstek J, 2017	−4.1	4.4	42	−1.6	4.4	83	4.0%	−0.56 [−0.94, −0.19]	2017
Jane M, 2017	−3.5	3.8	22	−1.8	2.1	18	3.3%	−0.53 [−1.16, 0.11]	2017
Gilmore LA, 2017	−0.1	0.9	19	1.8	0.9	16	2.8%	−2.06 [−2.90, −1.22]	2017
Spring B, 2017	−4.7	6.4	32	−6.6	6.4	32	3.7%	0.29 [−0.20, 0.79]	2017
Bender MS, 2017	−2.9	2.2	22	−0.3	2	23	3.3%	−1.22 [−1.86, −0.58]	2017
Bender MS, 2018	−1.1	1.2	33	−0.3	0.8	34	3.7%	−0.78 [−1.28, −0.28]	2018
Kliemann N, 2019	−4.5	10	17	−0.2	13.3	26	3.4%	−0.35 [−0.96, 0.27]	2019
Ozaki I, 2019	−3.7	2.8	24	0.6	2.9	22	3.2%	−1.48 [−2.14, −0.82]	2019
Lisón JF, 2020	−1.1	3	55	0.3	2.9	50	4.0%	−0.47 [−0.86, −0.08]	2020
**Total (95% CI)**			**1725**			**1544**	**100.0%**	**−0.66 [−0.88, −0.44]**	

Heterogeneity: $Tau^2 = 0.28$; $Chi^2 = 212.11$, df = 26 (P < 0.00001); $I^2 = 88\%$
Test for overall effect: Z = 5.89 (P < 0.00001)

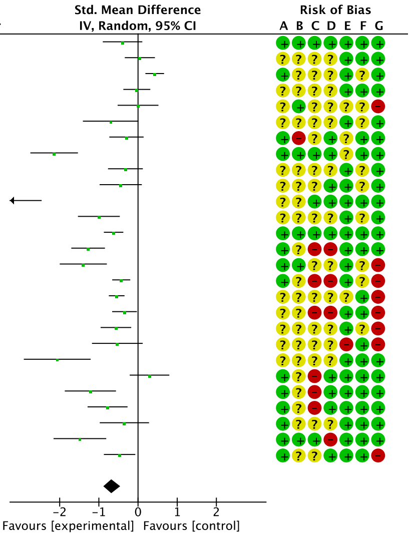

Risk of bias legend
(**A**) Random sequence generation (selection bias)
(**B**) Allocation concealment (selection bias)
(**C**) Blinding of participants and personnel (performance bias)
(**D**) Blinding of outcome assessment (detection bias)
(**E**) Incomplete outcome data (attrition bias)
(**F**) Selective reporting (reporting bias)
(**G**) Other bias

Supplementary Figure S7. Meta-analysis for mean weight change comparing web-based interventions with offline control groups for 3–6 months of duration. The forest plot shows standardized mean differences (SMD) for each article with 95% confidence intervals (CI). The diamond at the bottom of the graph means the meta-analyzed measure of effect and the lateral points of which indicate CIs for this estimate. A positive value reflects web-based interventions are more effective for weight loss than offline interventions. The area of each green square is proportional to the study's weight in the meta-analysis.

Study or Subgroup	web-based intervention Mean	SD	Total	Control Mean	SD	Total	Weight	Std. Mean Difference IV, Random, 95% CI	Year	A	B	C	D	E	F	G
Wylie-Rosett J, 2001	−4.7	1	183	−2.2	1.3	97	7.9%	−2.24 [−2.55, −1.93]	2001	?	?	?	?	+	?	+
Womble LG, 2004	−0.8	3.6	23	−3.3	4.1	24	6.7%	0.64 [0.05, 1.22]	2004	?	?	?	?	+	?	+
Adachi Y, 2007	−2.2	3	44	−1.4	2.4	50	7.5%	−0.29 [−0.70, 0.11]	2007	?	?	?	?	+	?	+
Werkman A, 2010	−1.9	3.1	166	−1.6	3	169	8.2%	−0.10 [−0.31, 0.12]	2010	+	?	−	−	+	+	−
Tanaka M, 2010	−2.4	3.2	23	−1.6	2.8	28	6.9%	−0.26 [−0.82, 0.29]	2010	?	?	?	?	+	?	+
Jebb SA, 2011	−6.7	6.5	230	−3.3	4.8	214	8.3%	−0.59 [−0.78, −0.40]	2011	+	+	−	−	+	+	+
van Wier MF, 2012	−1.9	5.7	450	−1.1	7	448	8.4%	−0.13 [−0.26, 0.01]	2012	+	+	?	?	+	?	−
Spring B, 2013	−4.5	6.5	29	−1	4.4	28	7.0%	−0.62 [−1.15, −0.09]	2013	+	?	?	?	+	+	−
Watson S, 2015	−4.4	3.6	25	−0.5	3.1	32	6.8%	−1.16 [−1.72, −0.59]	2015	+	+	+	+	+	+	−
Jakicic JM, 2016	−3.5	7.1	237	−5.9	7	233	8.3%	0.34 [0.16, 0.52]	2016	+	+	+	+	+	+	?
Phelan S, 2017	−3.2	5.4	174	−0.9	5.7	193	8.2%	−0.41 [−0.62, −0.21]	2017	?	?	?	+	+	+	−
Teeriniemi AM, 2018	−1.4	5	79	−0.4	4.6	68	7.9%	−0.21 [−0.53, 0.12]	2018	+	?	?	?	+	+	+
Thomas JG, 2019	−5.5	8.7	114	−6.4	10.3	56	7.9%	0.10 [−0.22, 0.42]	2019	+	+	?	+	+	+	−
**Total (95% CI)**			**1777**			**1640**	**100.0%**	**−0.38 [−0.71, −0.05]**								

Heterogeneity: Tau^2 = 0.34; Chi^2 = 244.53, df = 12 (P < 0.00001); I^2 = 95%

Test for overall effect: Z = 2.23 (P = 0.03)

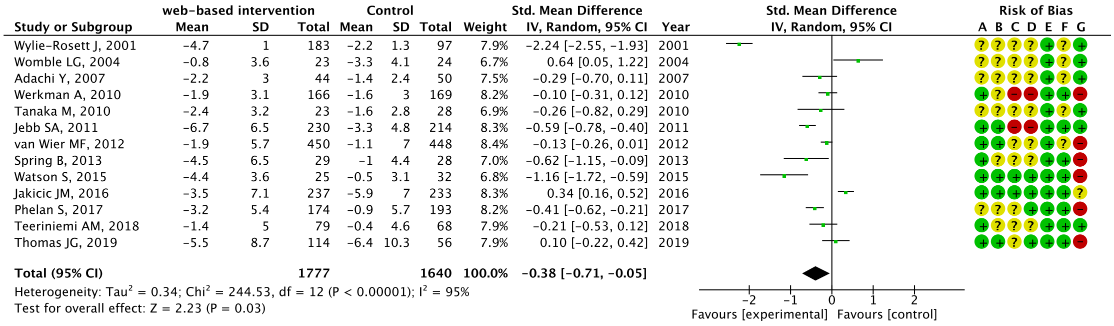

Risk of bias legend

(**A**) Random sequence generation (selection bias)

(**B**) Allocation concealment (selection bias)

(**C**) Blinding of participants and personnel (performance bias)

(**D**) Blinding of outcome assessment (detection bias)

(**E**) Incomplete outcome data (attrition bias)

(**F**) Selective reporting (reporting bias)

(**G**) Other bias

Supplementary Figure S8. Meta-analysis for mean weight change comparing web-based interventions with offline control groups for >6 months of duration. The forest plot shows standardized mean differences (SMD) for each article with 95% confidence intervals (CI). The diamond at the bottom of the graph means the meta-analyzed measure of effect and the lateral points of which indicate CIs for this estimate. A positive value reflects web-based interventions are more effective for weight loss than offline interventions. The area of each green square is proportional to the study's weight in the meta-analysis.
